# Children’s Diseases

**Published:** 1873-09

**Authors:** 


### CHILDREN'S DISEASES

have always a cause; they cannot be cured until the cause is removed, hence it is the parent's duty, in all cases of sickness, to take special pains to ascertain what made the child sick, not only as a necessary step towards present cure, but to prevent future sickness by avoiding the cause. But, if attention is given simply to remove the present sickness, it may occur again within a week, and very likely with greater force, because the system was debilitated by the first attack and has not as much power to resist disease.

Nine tenths of the ailments of nursing children arise from want of out door air, want of warmth, and want of suitable ood. Dress the baby warm; keep its little feet and legs and arms abundantly warm; give it out door air in some form every clear, bright, bracing day, and if it is sick at all it is because it is fed too often, or the food is unsuitable. When six months old the child should not be fed at shorter intervals than five hours—not an atom of food between; the mother's milk is best; next to that the milk of a healthy cow, the fresher the better, for the danger of souring, by being put in vessels not perfectly clean, is very great. The atmosphere begins to act unfavorably on milk within an hour after it is drawn, hence, if it could be given as soon as taken so much the better; it is clear that this was nature's intention. No substitute for the food of nursing infants ought ever to be used if cows' milk, freshly drawn, can possibly be had. It is inexcusable, it is criminal to give a sucking child any other food than its mother's milk, or that of a healthy cow, if it is possible to procure it. All the substitutes ever devised were expedients, in cases where cows' milk could not be had recently drawn, as in cities or on shipboard. Now and then a child may thrive on other forms of food, but they thrive in spite of it, not on account of its adaptiveness.

